# Direct non-productive HIV-1 infection in a T-cell line is driven by cellular activation state and NFκB

**DOI:** 10.1186/1742-4690-11-17

**Published:** 2014-02-07

**Authors:** Matthew S Dahabieh, Marcel Ooms, Chanson Brumme, Jeremy Taylor, P Richard Harrigan, Viviana Simon, Ivan Sadowski

**Affiliations:** 1 Biochemistry and Molecular Biology, University of British Columbia, Vancouver, BC V6T1Z3, Canada; 2 Department of Microbiology, The Global Health and Emerging Pathogens Institute; Mount Sinai School of Medicine, 1468 Madison Avenue, Annenberg building 18-50, New York, NY 10029, USA; 3 Division of Infectious Diseases, Department of Medicine, Mount Sinai School of Medicine, New York, NY 10029, USA; 4 BC Centre for Excellence in HIV/AIDS, Vancouver, BC V6Z1Y6, Canada

**Keywords:** HIV-1, Latency, LTR, CMV, Promoter, eGFP, mCherry, Double-label, Silent-infection, NFκB

## Abstract

**Background:**

Molecular latency allows HIV-1 to persist in resting memory CD4+ T-cells as transcriptionally silent provirus integrated into host chromosomal DNA. Multiple transcriptional regulatory mechanisms for HIV-1 latency have been described in the context of progressive epigenetic silencing and maintenance. However, our understanding of the determinants critical for the establishment of latency in newly infected cells is limited.

**Results:**

In this study, we used a recently described, doubly fluorescent HIV-1 latency model to dissect the role of proviral integration sites and cellular activation state on direct non-productive infections at the single cell level. Proviral integration site mapping of infected Jurkat T-cells revealed that productively and non-productively infected cells are indistinguishable in terms of genomic landmarks, surrounding epigenetic landscapes, and proviral orientation relative to host genes. However, direct non-productive infections were inversely correlated with both cellular activation state and NFκB activity. Furthermore, modulating NFκB with either small molecules or by conditional overexpression of NFκB subunits was sufficient to alter the propensity of HIV-1 to directly enter a non-productive latent state in newly infected cells. Importantly, this modulatory effect was limited to a short time window post-infection.

**Conclusions:**

Taken together, our data suggest that cellular activation state and NFκB activity during the time of infection, but not the site of proviral integration, are important regulators of direct HIV-1 non-productive infections.

## Background

Integrated HIV-1 provirus transcribes messenger and genomic RNA to produce progeny virions. However, the HIV-1 promoter can also exist in an inactive state, and the subsequent lack of viral products allows latently infected cells to escape both immune surveillance and viral cytopathic effects (reviewed in [1-3]). Importantly, latent HIV-1 remains functional and can be reactivated by cellular activation, for example. This results in proviral transcription and production of new virions [4]. Thus, HIV-1 latency, which allows the virus to persist indefinitely during highly active antiretroviral therapy (HAART), is one of the most significant barriers to HIV-1 eradication.

HIV-1 latency is generally regarded as a product of proviral transcriptional silencing. Numerous silencing mechanisms have been characterized using *in vitro* latency models that require cellular activation and long-term culturing to identify and isolate latently infected cells. Given these requirements, the majority of known silencing mechanisms pertain to the progressive silencing of productive infections and the maintenance of a latent state. Nevertheless, known HIV-1 transcriptional silencing mechanisms include: 1) suboptimal T-cell activation, 2) low levels of transcriptional activator function, 3) restrictive chromatin structure at the site of integration, 4) transcriptional interference at the site of integration, 5) low pTEF-b (CDK9/Cyclin T1) levels, and 6) repressive HIV-1 LTR nucleosome positioning and histone post-translational modifications (reviewed in [1-3]).

Without the ability to identify latently infected cells early, and in the absence of activation stimuli, it is difficult to evaluate which HIV-1 transcriptional silencing mechanisms are critical for latency establishment in newly infected cells. Thus, we and others have recently developed double-labeled HIV-1 latency models that can detect both productive and non-productive proviral states early post-infection [5,6]. Application of these models to both cell lines and activated primary CD4+ T-cells suggests that direct non-productive infections (latency) actually represent the majority of HIV-1 infections [5,6]. This conclusion is further supported by other studies identifying silent/inducible infections early in infection [7,8]. Taken together, these studies provide significant support for the role of direct silencing in HIV-1 latency establishment, and highlight the importance of studying establishment mechanisms in newly infected cells.

In this study, we use our doubly fluorescent HIV-1 reporter [5] to directly evaluate potential mechanisms responsible for the formation of direct non-productive states in newly infected Jurkat T-cells. We focus on two highly variable HIV-1 transcriptional regulatory mechanisms: 1) proviral integration site, and 2) cellular activation state and NFκB signaling. First, we show that direct non-productive infections occur at all sites of integration, thereby excluding a role for viral integration site locations. Instead, the occurrence of non-productive infections was inversely correlated with cellular activation state and NFκB activity. Moreover, modulating NFκB levels at the time of infection, either by small molecules or NFκB subunit overexpression, was sufficient to alter the occurrence of non-productive infection in newly infected cells. Taken together, our data suggest that the cellular level of NFκB activity at the time of infection, rather than the site of viral integration, controls the establishment of HIV-1 latency in newly infected T-cell lines. These findings are of relevance to HIV-1 eradication strategies since they may point to putative targets for therapeutic interventions minimizing HIV-1 latency establishment rather than latency reactivation.

## Results

**Figure 1:**
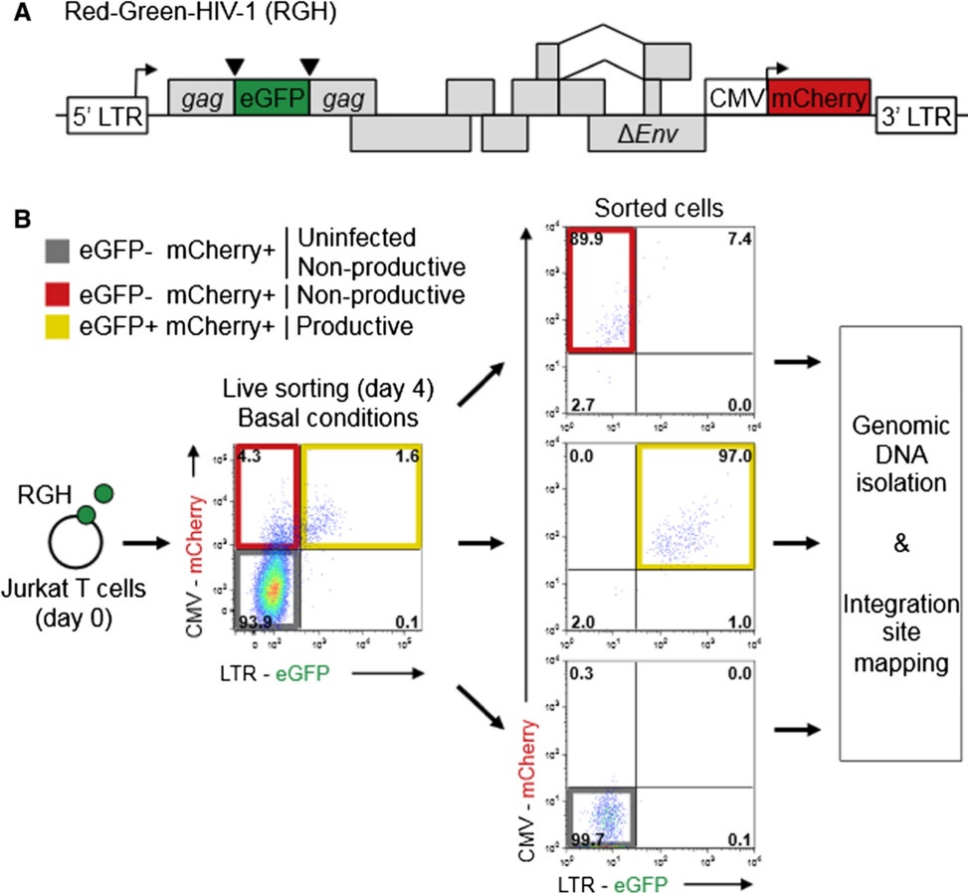
**Experimental outline for integration site mapping in newly infected cells. A**: Schematic representation of Red-Green-HIV-1 (RGH) used in this study. The virus contains a Gag fused eGFP marker under the control of the LTR promoter as well as a constitutively expressed mCherry marker under the control of the CMV_IE_ promoter inserted into the *nef* position. **B**: Schematic representation for isolation of productively and non-productively infected (RGH) Jurkat cells used for integration site mapping. Total RGH infected Jurkat cells were sorted based on eGFP and mCherry expression to greater than 90% purity four days post-infection. Genomic DNA from approximately 5 x 10^5^ cells of each sorted cell-population was isolated and used to map HIV-1 integration sites by 454 deep sequencing.

The doubly labeled Red-Green-HIV-1 (RGH) molecular clone is a recently described model that enables investigation of HIV-1 transcriptional regulatory mechanisms in newly infected, native state cells. This single-cycle vector incorporates both an LTR-driven gag-eGFP marker, and a CMV-driven mCherry marker in place of Nef, to allow for identification of both productively (eGFP+ mCherry+) and non-productively (eGFP- mCherry+) infected cells at single cell resolution (Figure 1A, [5]). We have previously used this vector to determine that the majority of HIV-1 proviruses are directly silenced shortly after infection in both cell lines and primary CD4+ T cells [5]. Since the majority of HIV-1 latency mechanisms described pertain to progressive epigenetic silencing, the determinants of direct non-productive infection remain unknown. In this study, we sought to use the recombinant RGH model to dissect the roles of proviral integration site and cellular activation state in regulating direct non-productive infection in Jurkat T-cells.

### Both productive and non-productive HIV-1 proviruses are integrated at similar locations

HIV-1 proviral integration sites are highly variable [1-3,9,10]. In some latency models, proximity to certain genomic features (alphoid repeats - [11], gene deserts - [12], and very highly expressed genes [7,12]) has been associated with proviral transcriptional silencing. However, a recent meta-analysis of integration sites found that, in five distinct latency models using either cell-lines or primary T-cells, these associations are not universal properties of HIV-1 latency, but rather are specific to the models in which they were identified [13]. Importantly, this study highlights the importance of characterizing the effect of integration site in each individual latency model. In this light, we sought to determine whether proviral integration sites were different between productively and non-productively RGH-infected Jurkat cells. We sorted total RGH infected cells into non-productively infected ‘red’ (eGFP- mCherry+; ~4% of total), and productively infected ‘yellow’ (eGFP+ mCherry+; ~2% of total) cell populations with more than 90% purity (Figure 1B). The ‘double negative’ population (eGFP- mCherry-) was also sorted and analyzed since we previously estimated that ~30% of all RGH infections result in direct repression of both the LTR and CMV promoters [5]. To identify sites of viral integration, genomic DNA from each population was extracted, digested with *MseI*, and ligated to adapters [14]. Nested PCR was used to amplify LTR-host chromosome junctions and resulting amplicons were sequenced by 454 pyrosequencing [14]. Reads were filtered for quality and mapped to the human genome using the INSIPID pipeline [15].

We mapped 2,900 and 4,271 unique integration sites in the ‘red’ and ‘yellow’ populations, respectively. Consistent with our previous characterization of ‘double negative’ RGH infected cells [5], we were also able to map 1,195 integration sites in this population, which represent proviruses in which both eGFP and mCherry markers were silenced directly upon infection.

**Figure 2:**
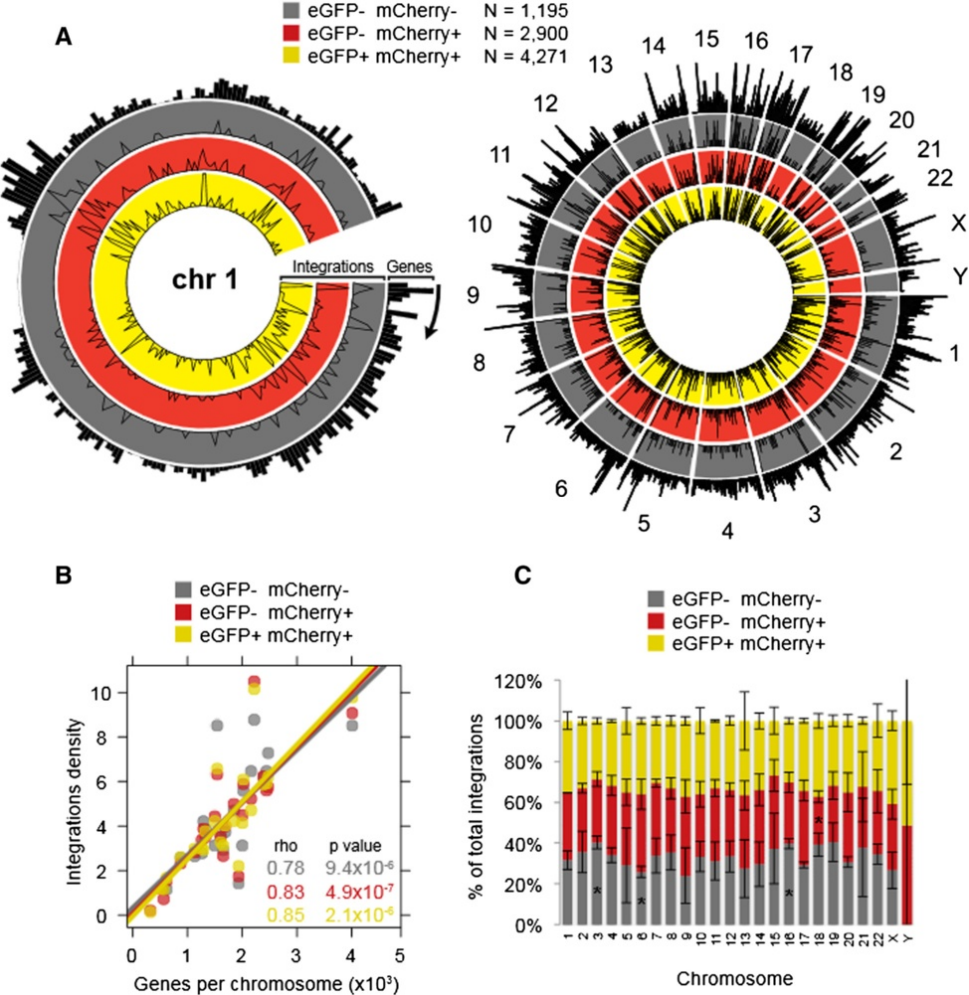
**Productive and non-productive RGH infections occur at similar viral integration sites across the genome. A**: Viral integration sites were mapped by 454 pyrosequencing in RGH infected Jurkat cells sorted for eGFP and mCherry expression. Data is shown for 1,195 eGFP- mCherry- integrations, 2,900 eGFP- mCherry+ integrations, and 4,271 eGFP+ mCherry+ integrations. Integrations and gene density across 1 Mb windows are plotted for chromosome one (left panel) and the entire genome (right panel). The outer track (black histogram) depicts gene density as annotated in the UCSC hg18 reference genome, while the inner tracks (colored line plots) indicate relative numbers of viral integrations. **B**: Integration density is plotted against gene density (genes per chromosome) for each sample. A linear regression line of the plotted points is shown. Samples were tested for significance by Spearman correlation. Rho coefficients and p values are listed. **C**: The proportion of total integrations in each chromosome is shown for each sorted cell population. Error bars represent standard deviations of triplicate experiments * p < 0.05 (Student’s T test).

To compare the integration sites between the cell populations, we first compared the density of integrations (1 Mb windows) across the whole human genome. High-resolution mapping of the integrations found within chromosome one, as well as within the entire human genome, revealed that in all cell populations integrations were largely constrained to gene dense areas, and that the integration densities in all three cell populations were largely overlapping (Figure 2A). Quantification of integration sites and gene densities showed a highly significant correlation for each population (Spearman correlation, ‘double negative’, rho = 0.78; ‘red’ , rho = 0.83; ‘yellow’ , rho = 0.85; p < 0.001 in all cases, Figure 2B). This preference for HIV-1 integrations in gene-rich areas is consistent with previous reports [9,10]. Quantification of integration density across the genome revealed similar integration site distributions for each of the ‘double negative’ , ‘red’ , and ‘yellow’ populations (Figure 2C). Of note, we did observe a minor but statistically significant decrease in integration density for the ‘red’ population in chromosome 18 (Figure 2C – p < 0.05). We also observed a modest but statistically significant increase in integrations into chromosomes 3 and 16, and a decrease in integrations into chromosome 6 for the ‘double negative’ population (Figure 2C - p < 0.05, ANOVA for number of integrations in each chromosome, and Student’s T test for number of 'double negative' integrations compared to 'red' and 'yellow' populations).

**Figure 3:**
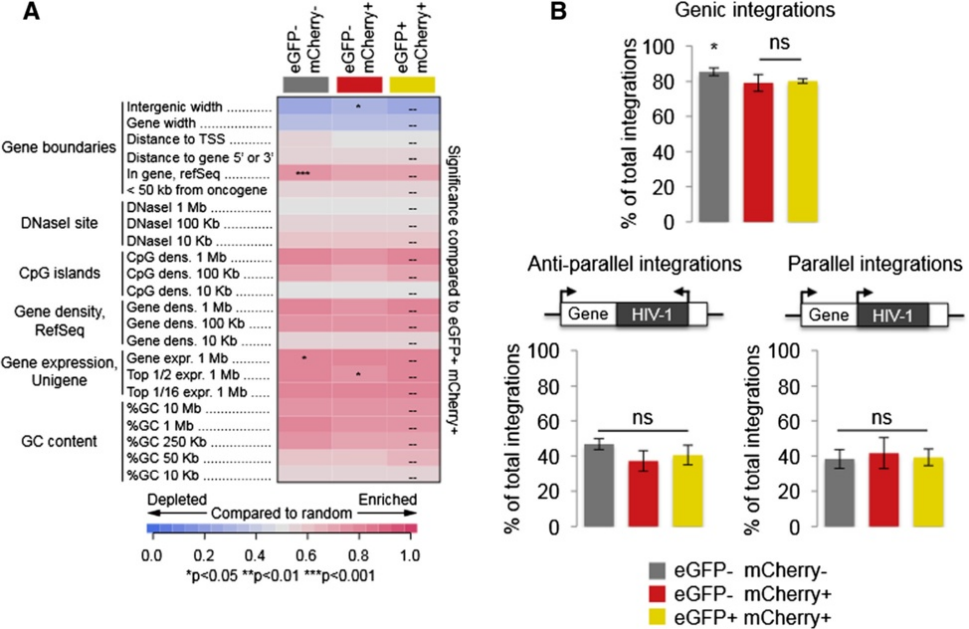
**Productive and non-productive RGH infections are indistinguishable in terms of genomic features at the site of integration, as well as proviral orientation. A**: Integration sites in each of the eGFP- mCherry-, eGFP- mCherry+, and eGFP+ mCherry+ samples were compared using the INSIPID heatmap tool for genomic features (Bushman Lab, University of Pennsylvania). Pink and blue colors represent enrichment and depletion of each feature, respectively, relative to matched random controls of integration sites. Statistical significance (ranked Wald tests) is shown relative to the eGFP+ mCherry+ population (dashes). **B**: The proportion of genic (top panel), anti-parallel (bottom left panel) and parallel (bottom right panel) integrations relative to total integrations is shown for each of the eGFP- mCherry-, eGFP- mCherry+, and eGFP+ mCherry+ infected cells. Error bars represent one standard deviation between triplicate experiments. ‘ns’ non-significant; * p <0.05 (Student’s T test).

High-throughput analysis of HIV-1 integration sites has previously revealed genomic preferences for HIV-1 integration in terms of gene density, distance to gene boundaries and transcriptional start sites (TSS), DNaseI hypersensitivity, CpG density, gene expression, and GC content [9,10]. Each of these characteristics are indicative of HIV’s preference to integrate into nucleosome-associated DNA within the introns of actively transcribed genes [9,10]. We used the INSIPID pipeline (Bushman Lab, University of Pennsylvania) to compare the genomic signatures of integration between each RGH infected population (Figure 3A). As a whole, the genomic properties of integrations in each population were consistent with previous studies [9,10], indicating that the RGH virus integrates into host chromatin similarly to wild type HIV-1. Importantly, the genomic signatures of the integration events in the ‘red’ population were highly similar to those in the ‘yellow’ population, indicating that they do not affect non-productive and productive infections (Figure 3A). Of note, we did observe a minor , but significant decrease in intergenic space (‘intergenic width’ – Figure 3A, p < 0.05, Wald test), and association with highly expressed genes (‘top ½ expr. 1 Mb Unigene’ – Figure 3A, p < 0.05, Wald test), suggesting that integrations in the ‘red’ population (non-productive infections) may be located in slightly less gene dense and less expressed regions than in the ‘yellow’ population. The profile of integrations in the ‘double negative’ population were similar to the ‘red’ and ‘yellow’ populations but the number of integrations into genes (coding or intronic sequence and irrespective of expression) was significantly increased (‘in gene, refSeq’ – Figure 3A, p < 0.001, Wald test; ‘gene expr. 1 Mb, Unigene’ – Figure 3A, p < 0.05). These findings suggest that, in certain contexts, integration into genes could repress transcription of both the LTR and CMV promoters in the RGH provirus (‘double negative’ population). Of note, the gene expression profiles used in this analysis were obtained from previous independent datasets produced with Jurkat cells [16].

We also compared the epigenetic landscape surrounding viral integration sites in the different RGH infected populations using INSIPID’s annotated epigenetic data from independent Jurkat and CD4+ T-cell experiments [15,17-23]. No significant differences were observed between the ‘red’ and ‘yellow’ populations (Additional file 1: Figure S1A). Integrations in the ‘double negative’ population were, however, significantly less frequently associated with nucleosomes and histone post-translational modifications, as compared to the ‘red’ or ‘yellow’ populations (Additional file 1: Figure S1A). This result, and the increased association with genes for the ‘double negative’ population (Figure 3A), suggests some effect of integration site on transcriptional repression. However, this effect is small and likely does not explain the transcriptional differences between the different RGH infected populations. Moreover, this is likely not an HIV-1 specific effect since both the LTR and CMV promoters are silenced in the ‘double negative’ population.

In human cells, the occurrence of transcriptional regulation-associated histone marks is often correlated with nucleosome position relative to gene promoters and gene bodies [17]. Therefore, we plotted RGH integration densities as a function of both the average distance across genes and the average distance from gene transcriptional start sites (TSS), however we observed no differences between cell populations in either case (Additional file 1: Figure S1B).

We next experimentally tested the effect of the epigenetic landscape on the productivity of RGH infection by utilizing an N74D capsid mutant that causes integration into regions of lower gene density and increased heterochromatin [24-26]. However, no differences were observed in the ratio of non-productive (‘red’) to productive (‘yellow’) infections between the RGH N74D capsid mutant and the wild-type RGH vector, further suggesting that epigenetic profiles surrounding integration sites are not major mediators of direct non-productive infection (Additional file 1: Figure S1C).

The orientation of proviral integrations within host genes has also been implicated in HIV-1 transcriptional regulation and latency [27-29]. However, another study did not observe a role for proviral orientation across multiple latency models [13]. Therefore, we compared the frequency of parallel and anti-parallel genic integrations between the RGH infected cell populations. The frequency of parallel and anti-parallel intragenic orientations were similar (~40% of total integrations), and not significantly different between cell populations (Figure 3B). Supplementary to proviral orientation, we analyzed the nucleotide sequences around the site of integration in RGH infected cells. These sequences were similar between cell populations and consistent with previously described HIV-1 target sites [9] (Additional file 2: Figure S2A). Moreover, gene ontology analysis did not reveal any differences in the types of genes harboring integrated provirus between the RGH infected cell populations (Additional file 2: Figure S2B).

Taken together, our data suggests that integration sites fail to play a significant role in regulating direct non-productive RGH infections in newly infected Jurkat cells. Therefore, alternative mechanisms are likely to dictate this process in this model T-cell system.

### Direct non-productive HIV-1 infection is associated with lower cellular activation and NFκB signaling

HIV-1 transcription is tightly linked to both cellular activation and the activity of signaling pathways downstream of the T-cell receptor (reviewed in [1,3]). Moreover, the NFκB pathway is an important and potent regulator of HIV-1 transcription (reviewed in [30]), and has been previously implicated in mediating early productive HIV-1 infections [7]. Given that direct non-productive RGH infection is independent of proviral integration sites (Figures 2 and 3, S1 and S2), we speculated that differences in cellular activation state and NFκB signaling around the time of infection could be responsible.

**Figure 4:**
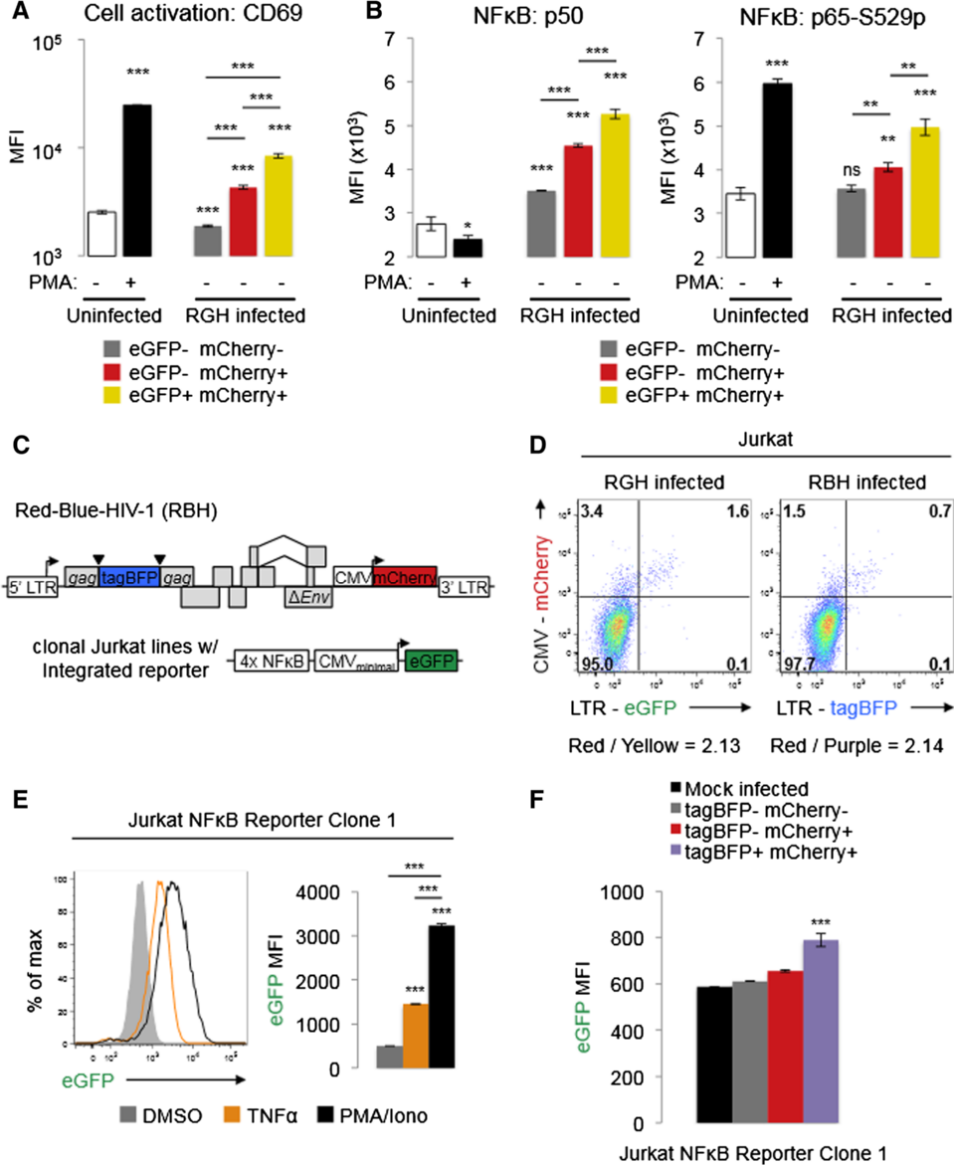
**Non-productive RGH infection inversely correlates with both cellular activation and NFκB activity. A**: RGH infected Jurkat cells (four days post-infection) were assayed for cellular activation by staining with anti-CD69 antibodies and analysis by flow cytometry. Uninfected cells were treated with either DMSO or PMA/Ionomycin for 24 hours prior to analysis. Error bars represent standard deviations of triplicate experiments. * p < 0.05, *** p < 0.001 (Student’s T test). **B**: Four days post-infection, RGH infected Jurkat cells were stained for NFκB p50 (left panel) and p65-S529-phospho (right panel) subunits and analyzed by flow cytometry. Uninfected cells were treated with DMSO or PMA/Ionomycin for 30 min prior to analysis. Error bars represent standard deviations of triplicate experiments. ‘ns’ non-significant, * p < 0.05, ** p < 0.01, *** p < 0.001 (Student’s T test). **C**: Schematic representation of Red-Blue-HIV-1 (RBH) and NFκB-eGFP reporter cell lines. RBH contains tagBFP in place of eGFP but is otherwise isogenic. Jurkat NFκB reporter cell lines contain a stably integrated eGFP marker driven by an NFκB responsive promoter (4x tandem NFκB *cis*-elements ‘GGGACTTTCC’ upstream of a CMV minimal promoter). **D**: Jurkat cells were infected with comparable amounts of RGH and RBH viral stocks. Cells were analyzed by flow cytometry four days post-infection. Plots shown are representative of multiple independent infection experiments. **E**: Jurkat NFκB reporter clone 1 was treated with DMSO, TNFα, or PMA/Ionomycin for 24 hours prior to analysis by flow cytometry for eGFP mean fluorescence intensity (MFI). Error bars represent one standard error of the mean. *** p < 0.001 (Student’s T test). **F**: Jurkat NFκB reporter clone 1 was infected with RBH and analyzed by flow cytometry at four days post-infection. Cells were gated into their constituent infected populations and then analyzed for eGFP MFI. Error bars represent one standard error of the mean. *** p < 0.001 (Student’s T test).

To analyze the effect of cellular activation on non-productive RGH infection we measured CD69 expression, a well characterized early T-cell activation marker [31], in the different populations of RGH infected cells. Staining total RGH infected cells for CD69 early post-infection (four days) showed that the gated ‘red’ and ‘yellow’ populations expressed approximately 2.3 and 4.5 fold more CD69 than mock-infected cells, respectively (Figure 4A). In contrast, the gated ‘double negative’ population (uninfected and eGFP- mCherry- infected cells) expressed CD69 at levels similar to the mock-infected cells (Figure 4A). Importantly, RGH infection itself did not activate Jurkat cells, as we did not observe substantial differences in CD69 expression between the total RGH infected population and mock infected cells (Additional file 3: Figure S3A). These data suggest that differences in LTR transcription from proviruses in newly infected cells are, in fact, associated with differences in cellular activation state.

To specifically address the role of NFκB in the establishment of direct non-productive infections, we infected cells with RGH and examined NFκB levels four days post-infection by intracellular staining for the DNA-binding p50 subunit of NFκB and the activated form of the trans-activating p65 subunit (S529-phospho) (Figure 4B). Both NFκB subunit levels were positively correlated with active transcription, as the gated ‘red’ and ‘yellow’ populations expressed approximately 1.3 and 1.5 fold more of both subunits, respectively, whereas the ‘double negative’ population expressed the lowest levels of both subunits (Figure 4B). Of note, expression of p50 and p65-S529-phospho increased concomitantly in 'red' and 'yellow' cells, suggesting that productive infections are associated with higher cellular levels of the activating form of NFκB (p65-p50) rather than the inhibitory p50-p50 form (Figure 4B). Importantly, RGH infection does not appear to up regulate NFκB, as the total RGH infected population and mock infected cells expressed similar amounts of both NFκB subunits (Additional file 3: Figure S3B).

To futher evaluate the role of NFκB signaling in promoting productive infection in newly infected cells, we simultaneously monitored both HIV-1 transcription as well as NFκB signaling at the single cell level. We created an RGH isogenic clone bearing the blue fluorescent protein tagBFP in place of eGFP (Red-Blue-HIV-1, RBH), as well as five Jurkat NFκB reporter cell lines bearing integrated eGFP constructs under the control of an NFκB responsive promoter (Figure 4C). Infection of Jurkat cells with RBH resulted in an infection profile similar to that of RGH i.e. the majority of RBH infections resulted in direct non-productive infection (Figure 4D). Treatment of the NFκB-eGFP reporter cell lines with known NFκB agonists TNFα and PMA/Iono resulted in 2.2 and 3.7 fold increases in eGFP mean fluorescence intensity (MFI), respectively (Figure 4E, Additional file 4: Figure S4A). Infection of the NFκB reporter cell lines with RBH virus showed that the productively infected cells (tagBFP + mCherry+, ‘purple’) were characterized by higher eGFP MFI (indicative of active NFκB signaling) compared to non-productively infected cells (tagBFP- mCherry+ ‘red’ , or tagBFP- mCherry- ‘double negative’ , Figure 4F – clone 1, Additional file 4: Figure S4B – clones 2–4). Importantly, RBH infection itself did not up regulate NFκB, as we did not observe substantial differences in eGFP fluorescence intensity between RBH- and mock-infected total cells (Additional file 3: Figure S3C). These data are consistent with the results of the intracellular NFκB staining of RGH infected Jurkat cells, and lend further support to the role of NFκB in regulating early RGH productive infections.

### NFκB modulating drugs administered at the time of infection can alter the occurrence of productive RGH infection

Our data indicate that cellular activation and NFκB signaling may influence the occurrence of direct non-productive infections in RGH infected cells (Figure 4). Therefore, we hypothesized that modulating NFκB activity during infection would affect the formation of direct non-productive infections. To test this, we treated Jurkat cells with TNFα (NFκB signaling agonist), BMS-345541 (IκB kinase inhibitor), SAHA (HDAC inhibitor) or DMSO (control) during RGH infection. The infected cells were cultured for three days, treated with either DMSO or PMA/Iono for 24 hours, and then analyzed by flow cytometry.

**Figure 5:**
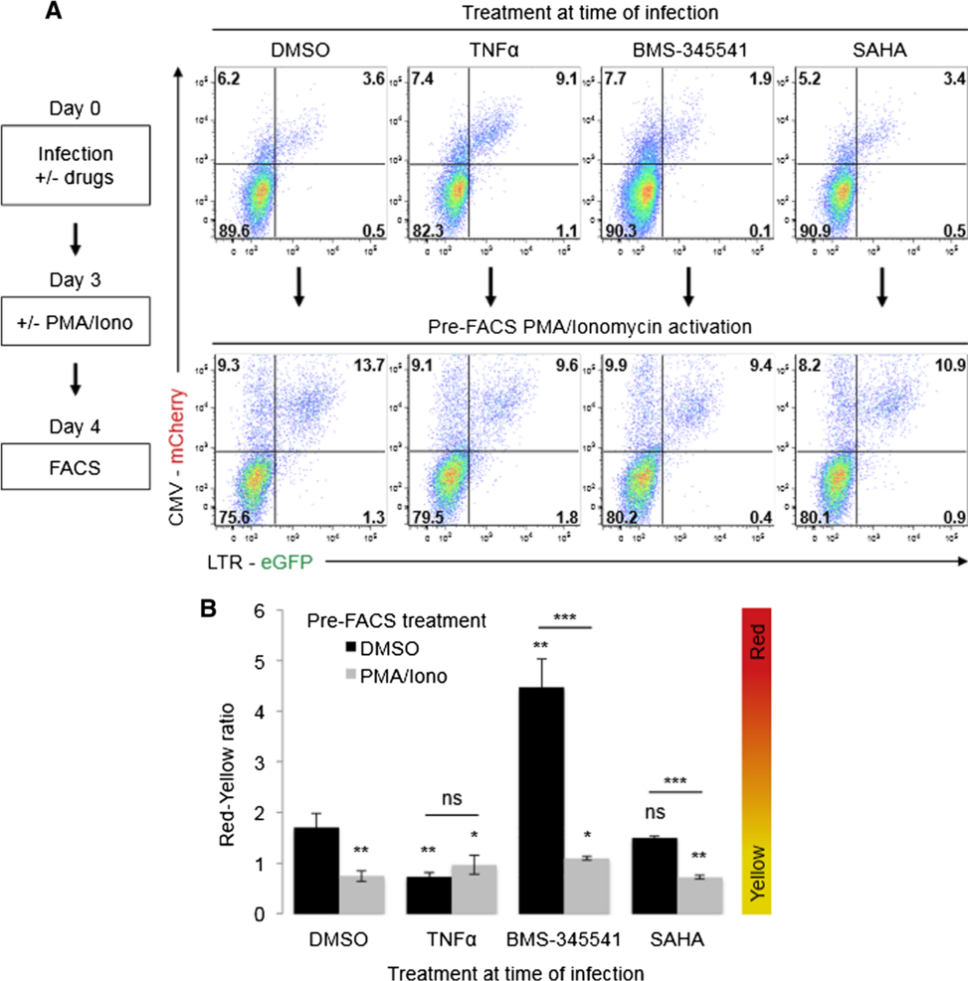
**The frequency of non-productive RGH infection can be altered by treatment of cells with NFκB modulating drugs at the time of infection. A**: Jurkat cells were treated with DMSO, TNFα, BMS-345541, or SAHA at the time of RGH infection. Four days post-infection cells were treated with either DMSO or PMA/Ionomycin for 24 hours and analyzed by flow cytometry. Plots shown are representative of triplicate infection experiments. **B**: Data from panel A is enumerated as the red-yellow ratio of infected cells. Error bars represent standard deviations of triplicate experiments. ‘ns’ non-significant, *** p < 0.001 (Student’s T test).

Cells treated with the DMSO control at the time of infection showed the typical higher frequency of non-productively infected cells compared to productively infected cells (‘red-yellow ratio’ ~1.7 – Figures 5A and B). Subsequent treatment with PMA/Iono prior to flow cytometry strongly induced LTR expression, resulting in an increase in productively infected cells (‘red-yellow ratio’ ~0.7, Figures 5A and B). In contrast, TNFα treatment at the time of infection reduced the number of non-productively infected cells by ~2.3 fold (‘red-yellow ratio’ ~0.7), indicating that NFκB up regulation during infection largely mitigates the formation of direct non-productive infection. Furthermore, TNFα treatment at the time of infection prevented LTR transcriptional silencing days later, as PMA/Iono treatment prior to analysis had no further effect in reducing the proportion of non-productively infected cells (Figures 5A and B). TNFα treatment at the time of infection increased the total number of both productive and non-productive infections, suggesting that proviruses present in the ‘double negative population’ were shifted to productive (‘yellow’) and non-productive (‘red’) infections (compare DMSO to TNFα treatment, Figure 5A). Of note, similar effects were observed with PMA/Iono pre-treatment which, in addition to other effects, stimulates NFκB activity in cells (data not shown). Conversely, down regulation of NFκB by BMS-345541 treatment at the time of infection resulted in a ~2.7 fold increase in the frequency of non-productive infections (‘red-yellow ratio’ ~4.4, Figure 5A and B). This increase in non-productive infections was counteracted by subsequent PMA/Iono treatment prior to flow cytometry, which increased productive infections by ~4.1 fold (‘red-yellow ratio’ ~1.1, Figure 5A and B). Interestingly, treatment with the HDAC inhibitor SAHA did not mitigate the formation of direct non-productive infection (‘red-yellow ratio ~ 1.5, Figure 5A and B), despite being a known activator of LTR transcription in other experimental settings [32,33]. This is consistent with previous reports [7], and suggests that epigenetic modifications, such as acetylation (sensitive to the HDAC1 inhibitor SAHA), are likely not a major mediator of direct non-productive infections. Indeed, SAHA (and HDAC inhibitors in general) modulates approximately 10-20% of genes non-specifically without affecting T cell receptor pathways [34].

Taken together, these results suggest that direct non-productive RGH infection is regulated by the action of NFκB signaling at the time of infection and that the propensity to form a non-productive infection can be modulated by NFκB agonists (TNFα and PMA/Iono) and antagonists (BMS-345541).

### Specifically modulating NFκB is sufficient to modulate the occurrence of productive RGH infection

While the major target of TNFα signaling is NFκB, TNFα can also affect the stress response related JNK-MAPK pathway and its downstream factor AP-1 (reviewed in [35]). Although TNFα-mediated reduction of RGH latency can likely be attributed to NFκB (Figures 4 and 5), it is possible that other pleiotropic effects may be contributing to the observed results. To test NFκB signaling in a more specific and temporal fashion, we generated Jurkat cell-lines bearing doxycycline inducible versions of a dominant negative (DN) form of the IκBα repressor (S32A/S36A - [36]), or the NFκB p65 subunit to allow direct down- or up-regulation of NFκB signaling, respectively.

**Figure 6:**
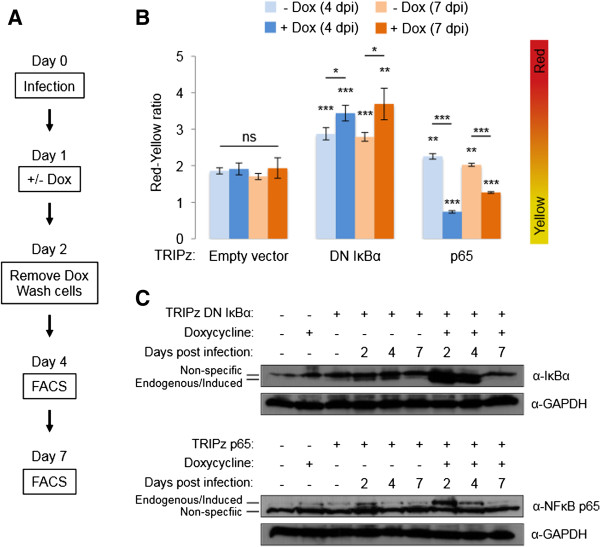
**Direct alteration of NFκB activity near the time of infection is sufficient to modulate the frequency of non-productive RGH infection. A**. Experimental outline for doxycycline-inducible NFκB modulation and RGH infection of Jurkat cells. **B**: Jurkat cells bearing a stably integrated, doxycycline-inducible expression construct containing either a dominant negative IκBα or p65 open reading frame were infected with RGH. Cells were cultured with or without doxycycline for 24 hours, and then washed and cultured in fresh media until analysis by flow cytometry at four and seven days post-infection. Error bars represent standard deviations of triplicate experiments. ‘ns’ non-significant, * p < 0.05, ** p < 0.01, *** p < 0.001 (Student’s T test). **C**: Immunoblot analysis of cells used in panel B. Jurkat whole cell extracts were separated by SDS-PAGE, transferred to nitrocellulose membrane, and blotted with antibodies against IκBα or p65.

We infected Jurkat cells containing the doxycycline inducible constructs with RGH and cultured the cells in the presence of doxycycline for 24 hours (day 1 to day 2 post-infection). Cells were washed and cultured in fresh complete media until flow cytometry at four and seven days post-infection (Figure 6A). Expression of DN IκBα resulted in a ~1.8 fold increase in the occurrence of non-productive infections, relative to empty vector control cells (‘red-yellow ratio’ ~3.4, Figure 6B). Of note, cells expressing the DN IκBα construct showed a ~1.5 fold increase in non-productively infected cells even in the absence of doxycycline (‘red-yellow ratio’ ~2.9, Figure 6B), which suggests leaky expression of the DN IκBα construct. In contrast to DN IκBα, cells expressing the p65 expression construct resulted in a ~2.6 fold decrease in the proportion of non-productively infected cells, relative to the empty vector control (‘red-yellow ratio’ ~0.7, Figure 6B). Importantly, these changes in productively and non-productively infected cells persisted even when cells were cultured in the absence of doxycycline for an additional three days (day 7 post-infection, Figure 6B). These results suggest that modulating NFκB near the time of infection exerts a lasting effect on the establishment of direct non-productive infections and that the observed effects are not due to continual modulation of NFκB signaling. To further test this, we measured IκBα and p65 levels by immunoblotting at two, four, and seven days post infection. Concurrent with the end of doxycycline treatment, we observed a transient increase in IκBα and p65 protein levels at day two post-infection (Figure 6C). This increase was specific to cells containing the expression construct and treated with doxycycline (Figure 6C). IκBα and p65 protein levels decreased substantially in the absence of doxycycline treatment four days post-infection, and returned to baseline at seven days post-infection (Figure 6C). Importantly, cells bearing the DN IκBα and p65 expression constructs still showed a significant change in the occurrence of productively and non-productively infected cells (‘red-yellow-ratio’) seven days post-infection (Figure 6B). This further supports the idea that modulating NFκB activity near the time of infection alters the fate of productive RGH infection on a permanent basis.

### The determination of productive RGH infection occurs around the time of infection

**Figure 7:**
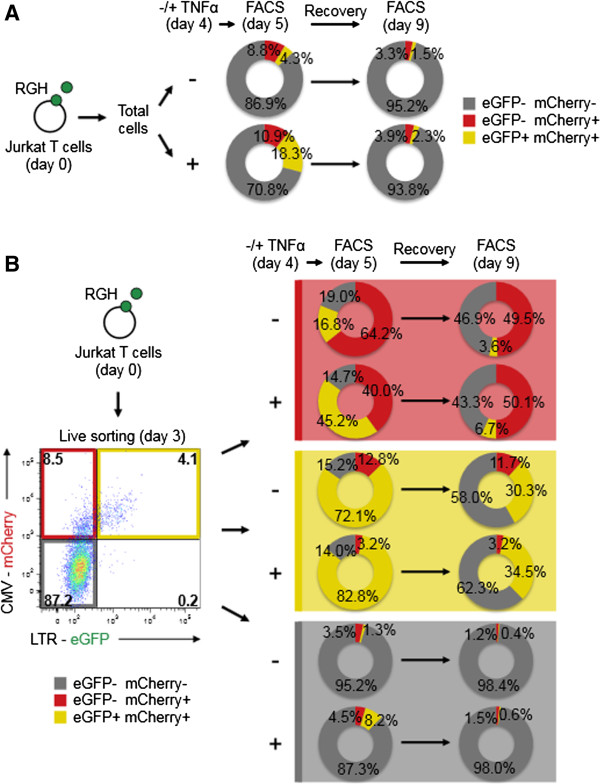
**The productivity of RGH infection is determined within four days post-infection. A**: Jurkat cells were infected with RGH. Four days post-infection, cells were treated with either DMSO or TNFα for 24 hours before analysis by flow cytometry. Cells were left to recover for four days prior to re-analysis by flow cytometry. The percentage of cells in each of the eGFP- mCherry-, eGFP- mCherry+, and eGFP+ mCherry+ quadrants is shown in the circle plots. Data shown is representative of duplicate experiments. **B**: Jurkat cells were infected with RGH and sorted three days post-infection into eGFP- mCherry-, eGFP- mCherry+, and eGFP+ mCherry+ (>90% purity). After 24 hours, the sorted cells were treated with either DMSO or TNFα for 24 hours prior to analysis by flow cytometry. Treated cells were left to recover for four days prior to further flow cytometry analysis. Data are representative of duplicate experiments.

Modulating NFκB activity at the time of infection altered the proportion of non-productive RGH infections days later (Figures 5 and 6). Therefore, we wanted to determine the time frame in which infection productivity is amenable to permanent modification by TNFα treatment. We reasoned that if a window of opportunity existed to alter the infection productivity, treatment of cells with TNFα outside of this window should only have transient effects on the productivity of RGH infection. To test this, we infected cells with RGH and treated them with either DMSO or TNFα at four days post-infection rather than at the time of infection. 24 hours post TNFα treatment, a portion of cells were analyzed by flow cytometry (to check for LTR induction), while the remaining cells were allowed to recover for another four days. Similar to TNFα treatment at the time of infection (Figures 5A and B), treatment with TNFα four days post-infection was able to reactivate a large proportion of non-productive proviruses, as demonstrated by a decrease in the size of the ‘red’ population, and a corresponding increase in the number of ‘yellow’ cells (Figure 7A). However, after a four day recovery, the DMSO and TNFα treated samples were largely indistinguishable in terms of the sizes of the ‘red’ and ‘yellow’ populations, suggesting that TNFα treatment at day four post-infection had not permanently altered the proportion of non-productively infected cells (Figure 7A). This data stands in contrast to the effect of treating cells with TNFα at the time of infection (Figures 5A and B), which suggests that the formation of direct non-productive infection occurs during, or shortly after, proviral integration.

To further explore the timing of RGH infection productivity, and to minimize the confounding impact of viral state on cellular outgrowth in a mixed population, we repeated the TNFα-treatment-recovery experiment with RGH infected Jurkat cells sorted into their constituent ‘double negative’ , ‘red’ , and ‘yellow’ subpopulations. In each of the ‘double negative’ and ‘red’ populations, TNFα treatment of sorted cells activated a substantial proportion of non-productive proviruses, as reflected in the increase in the number of ‘yellow’ cells (Figure 7B). As expected, TNFα treatment of the ‘yellow’ population had a minimal effect, as the majority of proviruses were already transcriptionally active (Figure 7B). Interestingly, when the sorted populations were left to recover for four days, the ‘double negative’ , ‘red’ , and ‘yellow’ TNFα treated cells all became indistinguishable from their matched DMSO treated pairs (Figure 7B). This data is consistent with results from the bulk RGH infected cells (Figure 7A). Of note, we did not observe major differences in the ratio of live cells (FSC/SSC) between DMSO and TNFα treatments, suggesting that HIV-induced cell-toxicity is not a substantial issue (data not shown). Furthermore, these findings collectively support the idea that non-productive RGH infection is established early (within four days post-infection) and permanently, such that TNFα treatment applied after the infection can no longer permanently alter the proportion of non-productively infected cells.

## Discussion

Despite extensive knowledge of individual mechanisms of HIV-1 transcriptional regulation, our understanding of the critical determinants for HIV-1 latency establishment in newly infected cells is limited. This knowledge gap is largely due to the inability to accurately identify latently infected cells early post-infection, and in their native state (i.e. without inducing cellular and viral activation). To circumvent these road-blocks, we and others have recent developed ‘double-labeled’ HIV-1 vectors incorporating constitutive markers of infection [5,6]. Initial studies with these models have revealed that a large proportion of HIV-1 infections result in a direct latent state, however the mechanisms by which these infections form remains unknown. In this study we used a doubly labeled HIV-1 latency model [5] to show that the cellular activation state and NFκB activity around the time of infection, but not viral integration site, are important for regulating direct non-productive infections in Jurkat T-cells.

Although primary CD4+ T-cells are considered to be the gold standard for HIV-1 latency models, we note a number of technical issues precluding the precise and unbiased evaluation of direct non-productive RGH infection in resting CD4+ T-cells. Nevertheless, we previously observed that RGH infection of activated primary CD4+ T-cells from three donors results in a high degree of direct non-productive infection, comparable to Jurkat cells [5]. Furthermore, another group also noted a high degree of latency in activated primary CD4+ T-cells [8]. This suggests that activated primary CD4+ T-cell infections may be accurately recapitulated in Jurkat cells. Thus, the results obtained with Jurkat cells in this study are likely to hold in primary T-cells, especially if primary cells must be activated in order to render them permissive to infection.

Previous reports have implicated integration site variability as a determinant for HIV-1 latency. Most notably, latency was correlated with integration into gene deserts, highly transcribed genes (high transcriptional interference) and alphoid repeats [11,12,37]. However, high-throughput analysis of HIV-1 integration sites indicates that integrations into such regions are highly disfavored [9,10]. Instead, most proviruses are located within actively transcribed genes that are enriched for histone marks associated with active chromatin (H3K4me3, lysine acetylation), and depleted for marks associated with repressive chromatin (H3K9me3, H3K27me3) [9,10,12,38,39]. We speculate that latency models using cellular activation and long-term culturing to identify and establish latency could select for the most strongly repressed latent proviruses, thereby resulting in an over-representation of such disfavored integration locations. Our analysis of proviral locations in RGH infected Jurkat cells shows little evidence for integration sites regulating the difference between ‘red’ (eGFP- mCherry+) and ‘yellow’ (eGFP+ mCherry+) cells (Figures 2 and 3, S1, and S2). Furthermore we did not find any evidence for enrichment of the aforementioned rare types of integration sites. Moreover, the frequency at which these types of integrations occur is incompatible with the degree of direct non-productive infections observed in the RGH model [5] and by other groups [6-8]. Our conclusions are in agreement with a recent meta-analysis of HIV-1 integration sites in five primary and cell line latency models [13]. In this study, the authors found no genomic predictors of latency and, interestingly, only little overlap of chromosomal features between latency models [13]. This highlights the intrinsic mechanistic variability of HIV-1 latency models, as well as the need to fully characterize determinants of latency in each model.

**Figure 8:**
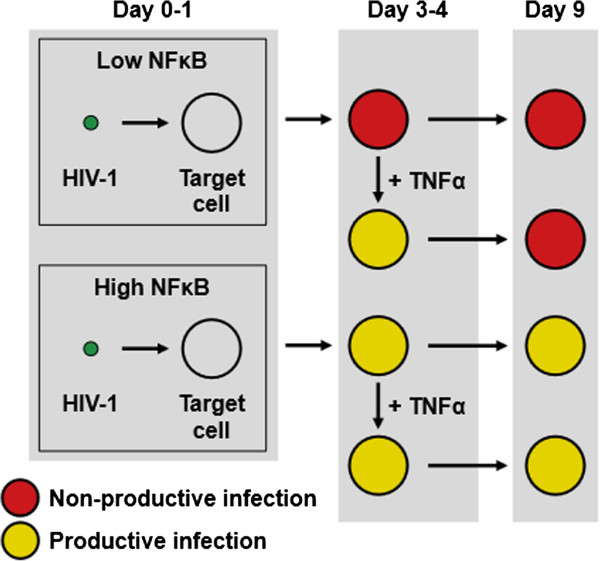
**Model of NFκB mediated effects on RGH productivity in newly infected cells.** Schematic representation of productive infection determination in RGH infected Jurkat cells. In cells with low NFκB activity, the majority of RGH infected cells exist in a non-productive state (eGFP- mCherry+, ‘red’) four days post infection, whereas cells with high NFκB activity have a greater propensity to exist in a productive state (eGFP+ mCherry+, ‘yellow’) and persist over time. TNFα stimulation of non-productively infected cells four days post infection only temporarily reactivates HIV-1, which reverts to its non-productive state days later.

In the absence of a role for integration sites in regulating direct non-productive RGH infection in newly infected Jurkat cells, the data presented in this study suggest that productive infection is positively correlated with cellular activation and NFκB activity (Figures 4, 5, 6, 7, and 8). Despite this understanding, it remains to be determined what drives fluctuations in cellular activity and corresponding NFκB activity within any given population of newly infected cells. In the physiological context of T-cells, the timing of infection during cellular deactivation (reversion from activated state to memory state) is a plausible driver of activation state heterogeneity [1]. Depending on the time of infection, cells may be at different points in the deactivation process and newly integrated proviruses may be exposed to highly variable T-cell signaling states and/or transcription factor pools. Complementary to this, thermodynamically driven stochastic fluctuations in cellular processes, which can drive phenotypic asymmetry in clonal cells (reviewed in [40,41]), may impact NFκB activity. Indeed, the NFκB and SP1 sites of the LTR have been implicated in controlling stochastic HIV-1 gene expression noise [42]. Other potential mechanisms of NFκB fluctuation include oscillatory behavior in response to TNFα signaling [43], and rapid nuclear shuttling of NFκB p65 and IκBα [44]. NFκB p65 shuttling was shown to provide low-level basal HIV-1 transcription in infected resting CD4+ T-cells [44], while shuttling of IκBα was shown to dampen leaky NFκB signaling by removing active NFκB p65 from the nucleus [44,45]. Taken together, the cumulative action of several sources of fluctuation could contribute to a wide spectrum of cellular activation and NFκB activity *in vivo.* Further work is needed to elucidate exactly what cellular state and NFκB activity level is deterministic for primary latency establishment.

Our data indicate that direct non-productive infections are established around the time of infection, and that this process is fundamentally different from latency in which productive infections are silenced over time (Figures 5, 6, 7, and 8). We note that treatments with NFκB agonists and antagonists early during infection could profoundly alter the occurrence of non-productive infection days later, whereas treatment four days post-infection did not result in long term modulation (Figures 7 and 8). This indicates that once a non-productive state is established during initial infection, it becomes ‘imprinted’, possibly through subsequent epigenetic modifications (Figure 8).

Our results contribute to an emerging body of work that links cellular activation state and transcription factor availability with the formation of HIV-1 latency. Although evidence is mounting that NFκB contributes to latency determination in newly infected cells (this study and [7]), we cannot exclude the actions of other transcription factors and/or upstream regulators in modulating latency. Most notably, the factors SP1 [42], AP1 [46], and the Jun N-terminal protein kinase (JNK) [47,48] have all been implicated in HIV-1 latency. It will be of great benefit to reconcile these studies and develop a comprehensive understanding of how individual factors/mechanisms act cumulatively to establish latency in newly infected cells. Indeed, fully understanding this process is paramount to successfully devising biologically relevant model systems suitable for screening novel latency modulating therapeutics.

## Conclusions

HIV-1 infection of Jurkat T-cells results in both productive and non-productive proviral states shortly after infection. Our data indicate that the differences between productive and non-productive infections are not caused by the location or orientation of viral integrations. Instead, the cellular activation state and NFκB activity around the time of infection determine the outcome of viral infections and, in turn, early latency.

## Methods

### Viral vectors and constructs

The Red-Green-HIV-1 (RGH) molecular clone was used as previously described [5]. To construct the gag-N74D RGH clone, the mutation was created by PCR mediated site directed mutagenesis and cloning of the amplicon into the *BspQI*/*ApaI* sites of the previously described RGH construct [5]. The Red-Blue-HIV-1 (RBH) molecular clone was created by cloning a synthesized tagBFP construct (GeneWiz) into the *SapI/SphI* sites of RGH.

pTRIPz-EV, pTRIPz-DN-IκBα and pTRIPz-p65 are derivatives of the commercial doxycycline-inducible lentiviral vector pTRIPZ-Ctrl (Thermo Fisher). pTRIPz-EV (empty vector) was created by digestion with *AgeI/MluI*, blunting with Klenow polymerase, and re-ligation. pTRIPz-DN-IκBα contains the S32A/S36A mutant version of the IκBα repressor PCR amplified from pSVK3-IKBα-2N [36], which was cloned into the *AgeI/MluI* sites of pTRIPz-Ctrl. pTRIPz-p65 contains a PCR amplified NFκB p65 open reading frame cloned into the *AgeI/MluI* sites of pTRIPz-Ctrl.

### Cell culture, virion production, and transduction

Jurkat E6-1 [49], HEK293T (ATCC), and derivative cell lines created in this study were cultured as previously described [5]. VSV-G pseudotyped viral stocks were created by transfecting HEK293T cells with envelope deleted viral molecular clones and pHEF-VSVg [50] in a 10:1 ratio as previously described [5]. Unless otherwise indicated, Jurkat E6-1 cells were spinoculated as previously described [5]. Briefly, 5 ×1 0^5^ cells in 1 mL culture media (+ 4 μg/mL polybrene) were spin-infected (1.5 hr, 500 × *g*, room temperature) with 25 μL of viral stock, so as to yield an average infection rate of than 10-15% and ensure single-copy integrations.

NFκB-eGFP viral stocks were produced in HEK293T cells by co-transfecting pGreenFire1-NF-κB (Systems Biosciences), pHEF-VSVg [50], pLP1-gag/pol, pLP2-Rev, and pcDNA3.1+-Tat (2 μg each, 30 μg polyethylenimine reagent). Purified and concentrated viral stocks were prepared as previously described [5]. NFκB reporter cell lines were created by transducing Jurkat cells with NFκB-eGFP virus (MOI ~ 4), followed by puromycin selection (1 μg/mL - Clontech). Resistant cells were subsequently maintained in complete media supplemented with 0.5 μg/mL puromycin.

pTRIPz viral stocks and stable cell lines were produced as described above for NFκB-eGFP, except that the lentiviral vectors pTRIPz-EV, pTRIPz-DN-IκBα or pTRIPz-p65 were used.

### Flow cytometry and staining

Analysis of infected cells by flow cytometry and live cell sorting were performed as previously described [5]. Of note, in all experiments, analysis was limited to live cells by FSC/SSC gating at the time of data acquisition. Unless otherwise stated, infected cells were analyzed four days post-infection. Jurkat E6-1 cells were stained and analyzed for CD69 as previously described [51] except that antibodies were conjugated to PE-Cy7 and 1 μL of antibody was used per 1 × 10^5^ cells (BD Biosciences). Jurkat cells were stained with PE-Cy7-NFκB p65 (pS529) (BD Biosciences) and NFκB p50 (Abcam) with Pacific Blue conjugated secondary antibody (Life Technologies) as previously described [52].

### Compound treatments

Infected cells were treated with the various compounds for the times and durations indicated in individual experiments. Compounds were added at the listed concentrations to complete media. Unless otherwise stated, compounds were used at the following concentrations: TNFα, 10 ng/mL (Sigma); SAHA, 0.5 μM [53]; PMA, 4 ng/mL (Sigma); Ionomycin, 1 μM (Sigma), BMS-345541, 5 μM (Sigma).

### Pyrosequencing of integration sites

HIV-1 integration sites were analyzed by 454 deep-sequencing as previously described [14]. Briefly, Jurkat cells were infected with RGH and sorted into constituent populations three days post-infection (eGFP- mCherry-, eGFP- mCherry+, eGFP+ mCherry+). Genomic DNA was extracted from ~ 5 × 10^5^ cells of each population, digested with *MseI* and ligated to adaptors. Nested PCR with adapter and LTR specific primers was performed to amplify the HIV-host genome junctions. After gel extraction of 100–600 bp fragments, amplicons were subjected to pyrosequencing on a 454 GS Junior machine (Roche). Data was analyzed using the Integration Site Pipeline and Database (INSIPID) web tool (Bushman Lab - http://microb215.med.upenn.edu/Insipid/ - [10,15], Circos [54], SeqMonk software (http://www.bioinformatics.babraham.ac.uk/projects/seqmonk/), WebLogo3 (http://weblogo.threeplusone.com/create.cgi), and the R/Bioconductor package ‘goProfiles’ (http://bioconductor.org/packages/2.11/bioc/html/goProfiles.html).

### Immunoblotting

RGH infected Jurkat cells were lysed in NP-40 lysis buffer (50 mM Tris, pH 8.0, 150 mM NaCl, 1% (v/v) NP-40, 0.1% (w/v) SDS) supplemented with 1x protease inhibitor cocktail (Roche). Lysates were cleared by centrifugation (10 min, 16000 × *g*, 4°C), mixed with 4× SDS-PAGE sample buffer, and boiled for 5 min. Whole cell extracts (40 μg) were separated on a 12% SDS-PAGE gel and then transferred to nitrocellulose membrane. Membranes were blocked with 2% (w/v) BSA in PBS-Tween (0.05% v/v) and then incubated with primary antibody overnight at 4°C. Antibodies used were as follows: IκBα – Abcam 32518 [1:5000], NFκB p65 – Abcam 7970 [1:500], GAPDH – Abcam 9484 [1:4000]. After washing and incubation with HRP conjugated secondary antibody, membranes were washed and signal was developed with SuperSignal West Femto chemi-luminescent substrate (Thermo Fisher).

### Statistical analysis

Unless otherwise stated, experiments were performed in biological triplicate. Where appropriate, statistical inference was performed on quantitative data. Two group testing was performed using the Student’s T-test, while comparison between multiple groups was made using one-way-ANOVA followed by pairwise two-group testing (Student’s T-test). Statistical analysis was performed in R 2.15.1 (http://www.r-project.org/). Integration site analysis heatmaps were created using the INSIPID pipeline (Figures 3A and Additional file 1: Figure S1B) utilizing previously described statistical methodology [10,15]. Briefly, for each identified integration site, matched random controls were created *in silico*. This pairing of experimental and control sites allows for computation of relative enrichment and de-enrichment profiles using a receiver operating characteristic framework. Comparisons between sets of integration sites (samples) for statistical significance are performed by calculating Wald-type test statistics, which are then tested using Chi Square methods.

## Competing interests

The authors declare that they have no competing interests.

## Authors’ contributions

MSD and MO conceived, designed and performed the experiments and analyzed the data. MSD, MO, VS and IS wrote the manuscript. CB, JT and PRH performed 454 deep sequencing. All authors read and approved the final manuscript.

## Supplementary Material

Additional file 1: Figure S1 Productive and non-productive RGH infections occur regardless of epigenetic properties at sites of integration A: Epigenetic properties of identified integration sites were compared between samples using the INSIPID heatmap tool for epigenetic features (Bushman Lab, University of Pennsylvania). Included features were limited to those identified in high-throughput studies of Jurkat and primary CD4+ T-cells. Yellow and blue colors represent depletion and enrichment of each feature, respectively, relative to matched random controls of integration sites. Statistical significance (ranked Wald tests) is shown relative to the eGFP+ mCherry+ population (dashes). B: Proviral integrations across the entire human genome are plotted as a function of the average distance across gene bodies (5’ to 3’ – top panel), and the average distance from gene transcriptional start sites (TSS – bottom panel). Data are plotted as pale filled circles with darker smoothed lines (Loess) overlaid. C: Jurkat cells were infected with equal amounts of wild-type RGH or an RGH version containing an N74D mutation in Capsid. Cells were analyzed by flow cytometry four days post-infection. Representative plots (left) and graphical quantitation (right) are shown. Error bars represent standard deviations of triplicate experiments. ‘ns’ non-significant. Click here for file

Additional file 2: Figure S2 Productive and non-productive RGH infections occur regardless of DNA sequence at the point of integration, or functional annotation of host genes. A: Weblogo3 analysis of the DNA sequence (+/- 20 bp) surrounding each integration site in the eGFP- mCherry- (‘double negative’), eGFP- mCherry+ (‘red’), and eGFP+ mCherry+ (‘yellow’) populations. B: Gene ontology analysis of integration sites in the eGFP- mCherry- (‘double negative’), eGFP- mCherry+ (‘red’), and eGFP+ mCherry+ (‘yellow’) populations, using *goProfiles.* Click here for file

Additional file 3: Figure S3 RGH infection does not induce cellular activation or NFκB signaling. A. Mock- and total-RGH-infected Jurkat cells were stained for CD69 four days post-infection. Data shown is representative of triplicate experiments. B. Mock- and total-RGH-infected Jurkat cells were stained for either NFκB p50 or NFκB p65-S529phospho four days post-infection. Data shown is representative of triplicate experiments. C. Mock- and total-RBH-infected Jurkat NFkB-eGFP reporter cell lines 1–5 were analyzed by flow cytometry four days post-infection. Data shown is representative of multiple experiments. Click here for file

Additional file 4: Figure S4 Characterization of RBH infection and Jurkat NFκB reporter cell lines. A: Jurkat NFκB reporter clones 2–5 were treated with DMSO, TNFα, or PMA/Ionomycin for 24 hours prior to analysis by flow cytometry for eGFP mean fluorescence intensity (MFI). Error bars represent one standard error of the mean. B: Jurkat NFκB reporter clones 2–5 were infected with RBH viral stock and analyzed by flow cytometry at four days post-infection. Cells were gated into their constituent infected populations and then analyzed for eGFP MFI. Error bars represent one standard error of the mean. Click here for file
