# Supplementary figures and images for: Direct non-productive HIV-1 infection in a T-cell line is driven by cellular activation state and NFκB
Source: Retrovirology. 2014 Feb 7;11:17. doi: 10.1186/1742-4690-11-17 (PMC4015675; doi:10.1186/1742-4690-11-17)

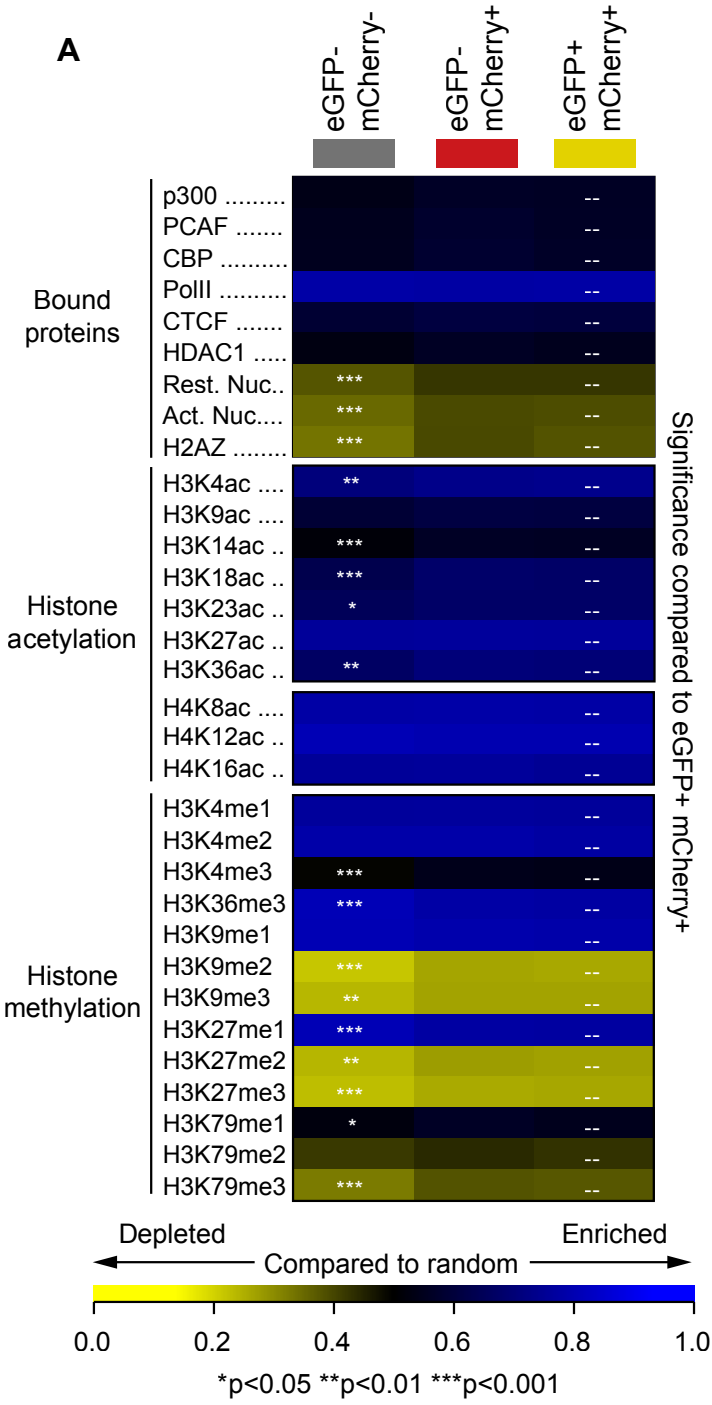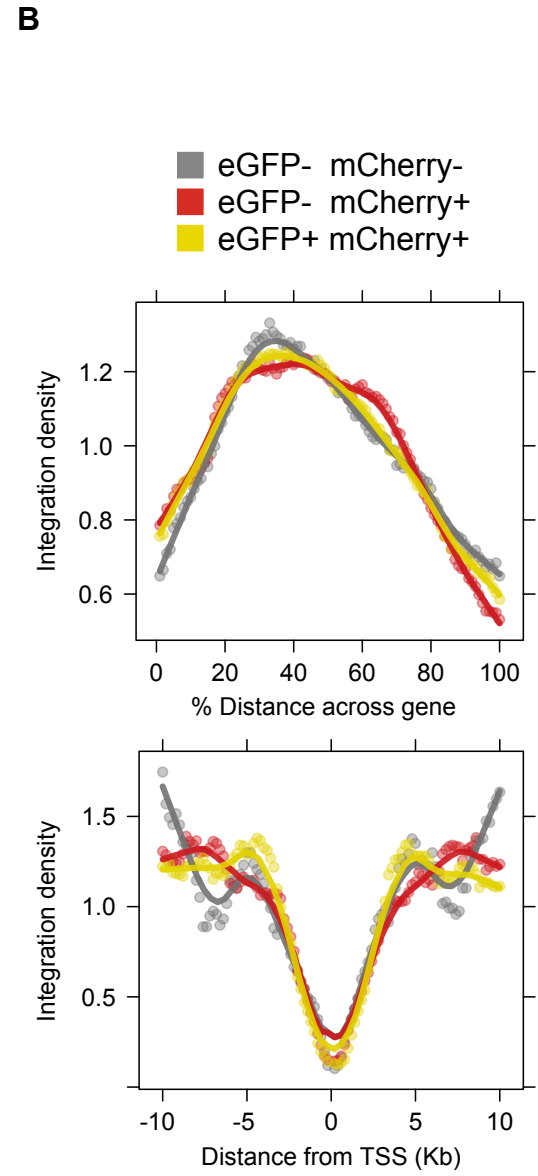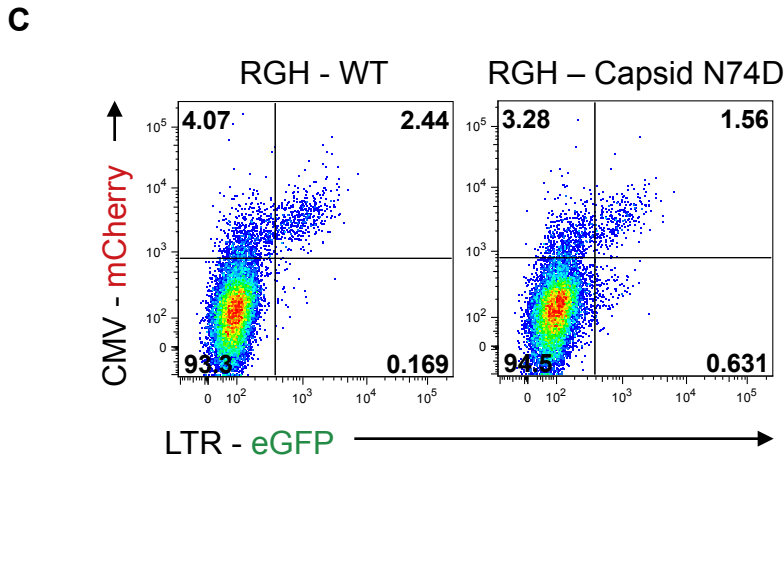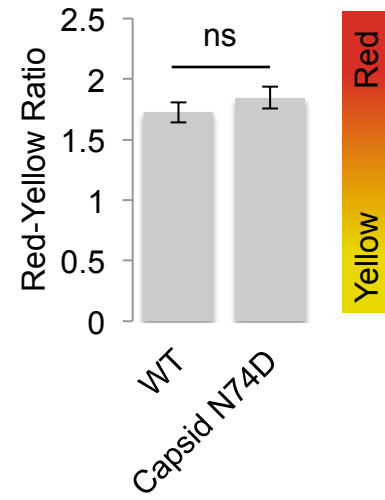

Supplement: Additional file 1: Figure S1 — Productive and non-productive RGH infections occur regardless of epigenetic properties at sites of integration A: Epigenetic properties of identified integration sites were compared between samples using the INSIPID heatmap tool for epigenetic features (Bushman Lab, University of Pennsylvania). Included features were limited to those identified in high-throughput studies of Jurkat and primary CD4+ T-cells. Yellow and blue colors represent depletion and enrichment of each feature, respectively, relative to matched random controls of integration sites. Statistical significance (ranked Wald tests) is shown relative to the eGFP+ mCherry+ population (dashes). B: Proviral integrations across the entire human genome are plotted as a function of the average distance across gene bodies (5’ to 3’ – top panel), and the average distance from gene transcriptional start sites (TSS – bottom panel). Data are plotted as pale filled circles with darker smoothed lines (Loess) overlaid. C: Jurkat cells were infected with equal amounts of wild-type RGH or an RGH version containing an N74D mutation in Capsid. Cells were analyzed by flow cytometry four days post-infection. Representative plots (left) and graphical quantitation (right) are shown. Error bars represent standard deviations of triplicate experiments. ‘ns’ non-significant. [file 1742-4690-11-17-S1.pdf]

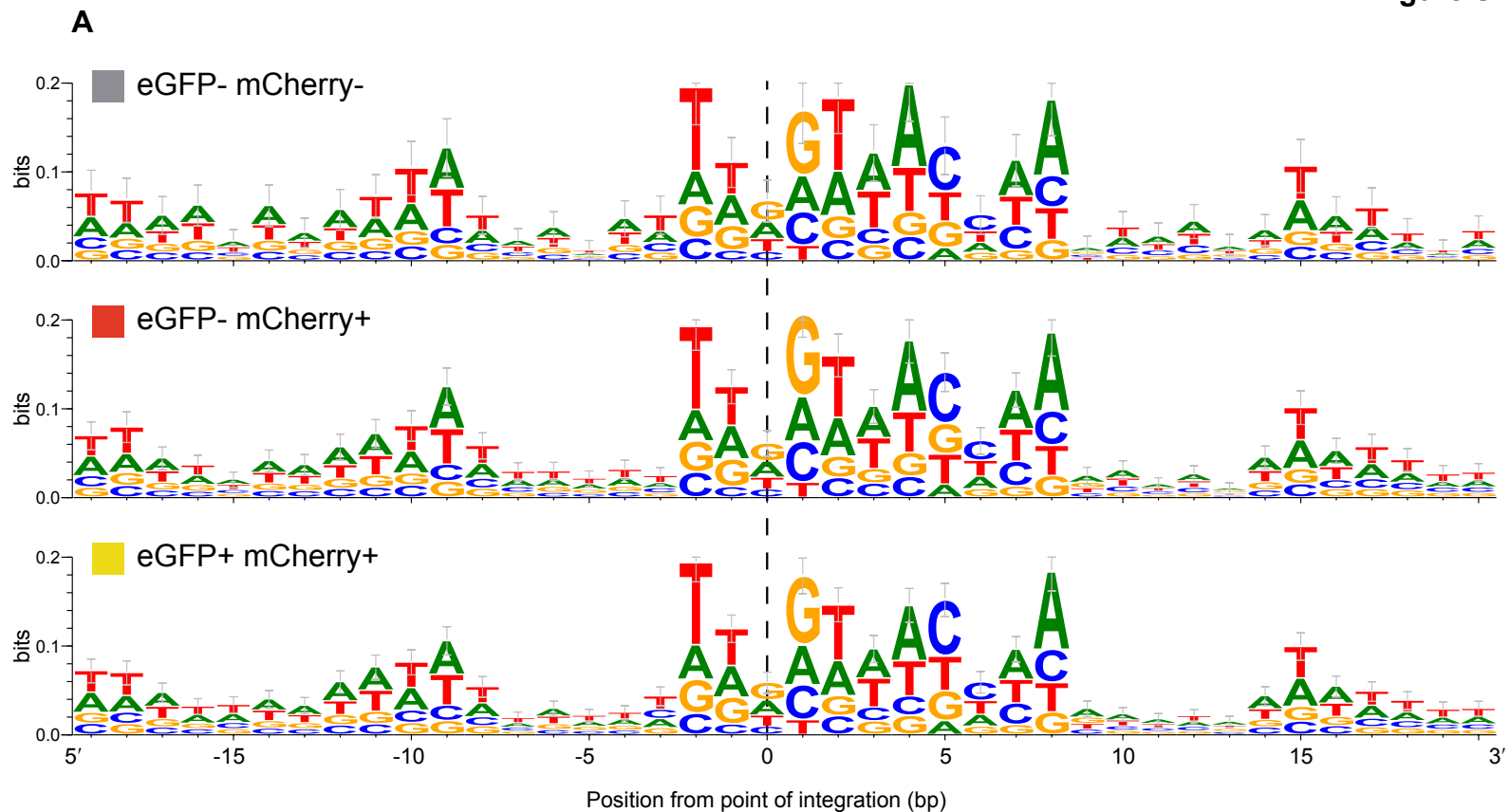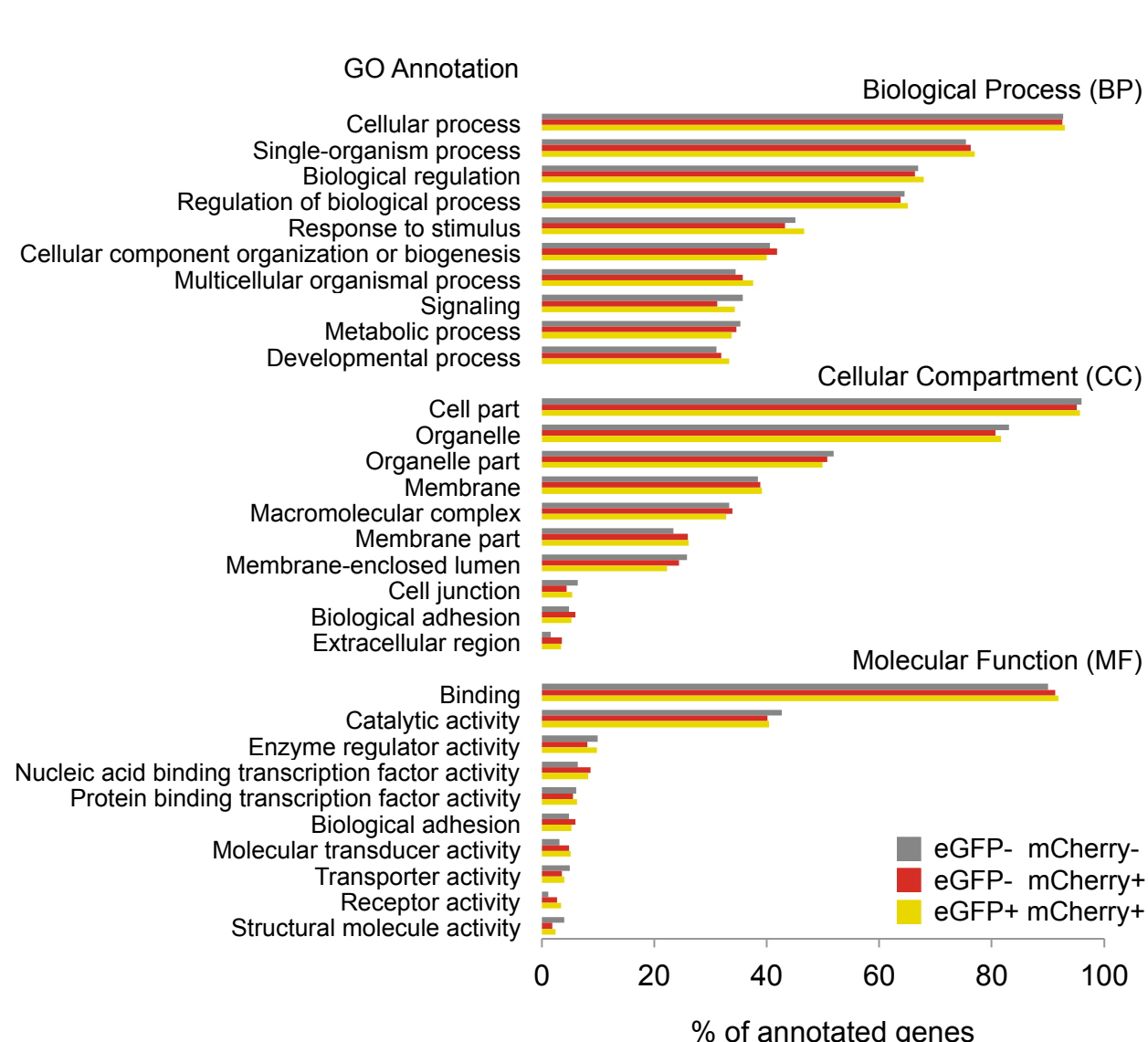

Supplement: Additional file 2: Figure S2 — Productive and non-productive RGH infections occur regardless of DNA sequence at the point of integration, or functional annotation of host genes. A: Weblogo3 analysis of the DNA sequence (+/- 20 bp) surrounding each integration site in the eGFP- mCherry- (‘double negative’), eGFP- mCherry+ (‘red’), and eGFP+ mCherry+ (‘yellow’) populations. B: Gene ontology analysis of integration sites in the eGFP- mCherry- (‘double negative’), eGFP- mCherry+ (‘red’), and eGFP+ mCherry+ (‘yellow’) populations, using goProfiles. [file 1742-4690-11-17-S2.pdf]

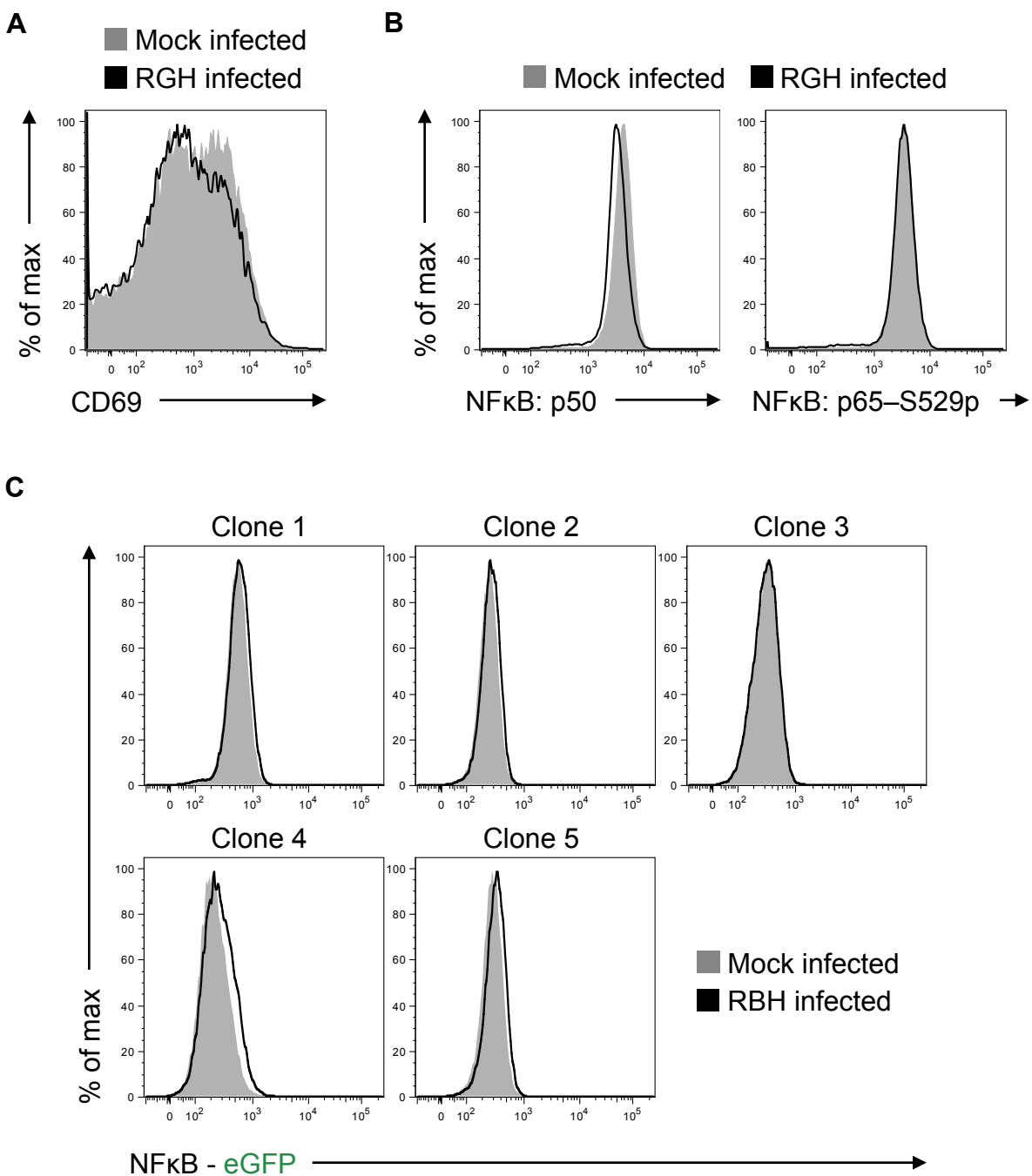

Supplement: Additional file 3: Figure S3 — RGH infection does not induce cellular activation or NFκB signaling. A. Mock- and total-RGH-infected Jurkat cells were stained for CD69 four days post-infection. Data shown is representative of triplicate experiments. B. Mock- and total-RGH-infected Jurkat cells were stained for either NFκB p50 or NFκB p65-S529phospho four days post-infection. Data shown is representative of triplicate experiments. C. Mock- and total-RBH-infected Jurkat NFkB-eGFP reporter cell lines 1–5 were analyzed by flow cytometry four days post-infection. Data shown is representative of multiple experiments. [file 1742-4690-11-17-S3.pdf]

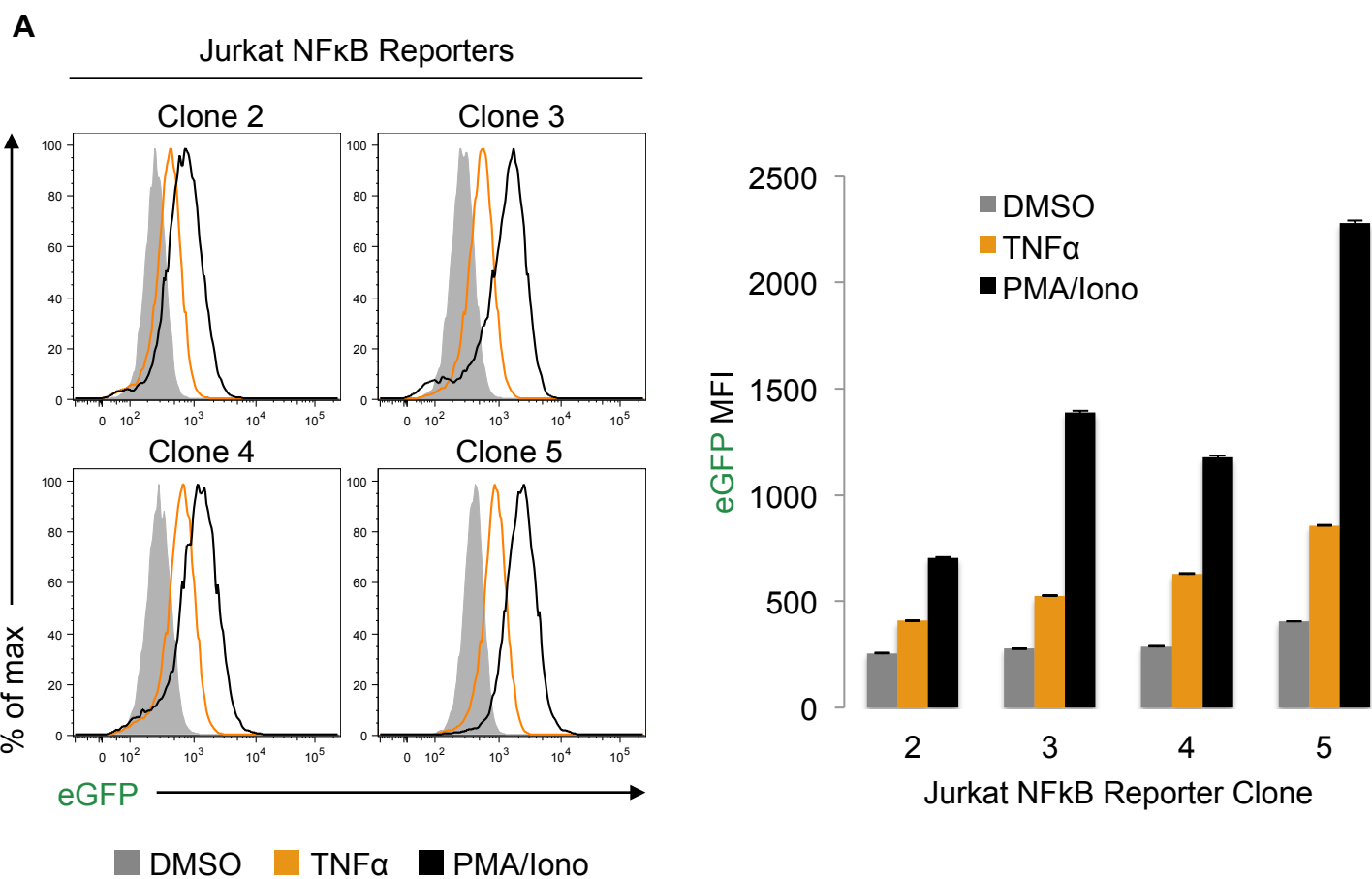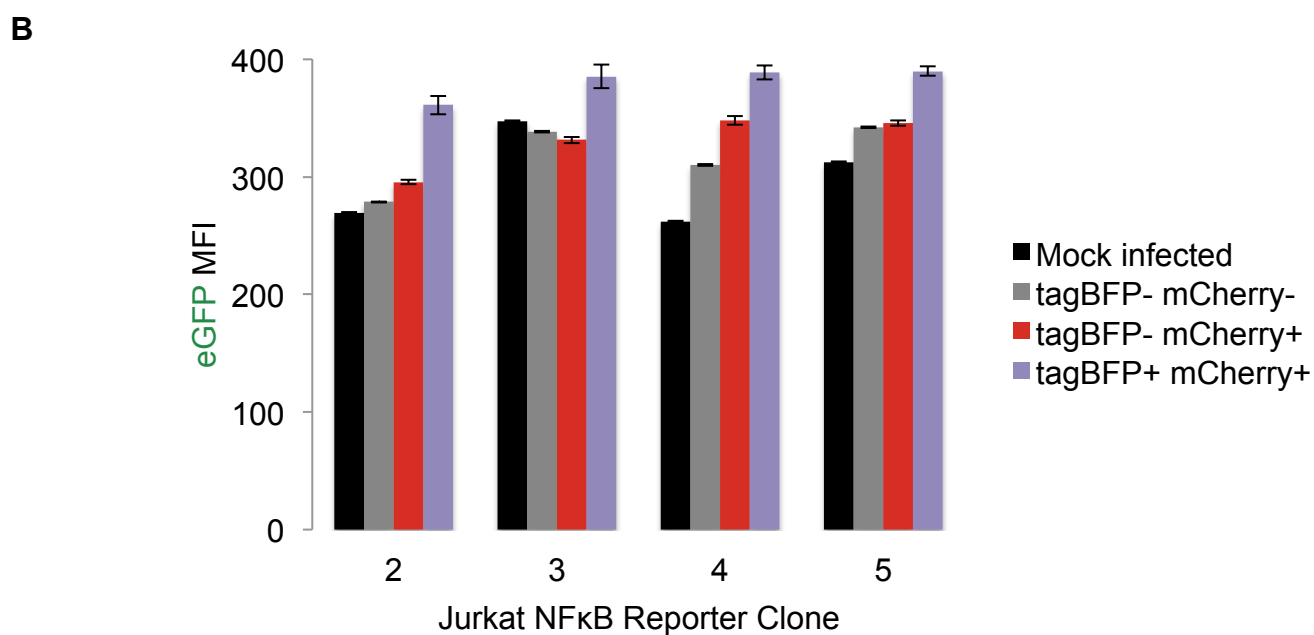

Supplement: Additional file 4: Figure S4 — Characterization of RBH infection and Jurkat NFκB reporter cell lines. A: Jurkat NFκB reporter clones 2–5 were treated with DMSO, TNFα, or PMA/Ionomycin for 24 hours prior to analysis by flow cytometry for eGFP mean fluorescence intensity (MFI). Error bars represent one standard error of the mean. B: Jurkat NFκB reporter clones 2–5 were infected with RBH viral stock and analyzed by flow cytometry at four days post-infection. Cells were gated into their constituent infected populations and then analyzed for eGFP MFI. Error bars represent one standard error of the mean. [file 1742-4690-11-17-S4.pdf]
